# Racial Disparities in Cardiometabolic Disorders Among Alzheimer's Disease Patients: A Study on Native Hawaiians and Pacific Islanders

**DOI:** 10.7759/cureus.76197

**Published:** 2024-12-22

**Authors:** Justin H Wong, Anna Gan, Lauren Nguyen, Lea Zoe El-Hage, Keao Kawaakoa, Meliza Roman, Chathura Siriwardhana, Enrique Carrazana, Kore K Liow

**Affiliations:** 1 Department of Medicine, University of Hawaii John A. Burns School of Medicine, Honolulu, USA; 2 Department of Neuroscience, Middlesex University, London, GBR; 3 Department of Integrative Biology and Physiology, University of California Los Angeles, Los Angeles, USA; 4 Department of Quantitative Health Sciences, University of Hawaii John A. Burns School of Medicine, Honolulu, USA; 5 Department of Neurology, University of Hawaii John A. Burns School of Medicine, Honolulu, USA; 6 Department of Neurology, Hawaii Pacific Neuroscience, Honolulu, USA

**Keywords:** alzheimer disease, cardiometabolic disorder, diabetes, native hawaiian, pacific islander

## Abstract

Background: Cardiometabolic disorders may accelerate the progression of Alzheimer's disease (AD), potentially impacting ethnic-racial groups with a higher prevalence of diabetes, obesity, and cardiovascular disease, though limited data exists on Native Hawaiians and Pacific Islanders (NHPI) populations.

Objective: This study aims to examine the prevalence of diabetes and associated comorbidities among AD patients from different ethnic-racial groups - Asians, Whites, and NHPIs - in Hawaii, with a focus on identifying risk factors linked to AD.

Method: A retrospective review was conducted on AD patient records from a single center in Hawaii, spanning June 2018 to June 2024. Variables assessed included age at diagnosis, sex, race, insurance type, alcohol use, comorbidities, and Mini-Mental State Examination (MMSE) scores. Statistical comparisons were conducted to identify group differences.

Results: Among 540 patients (256 Asians, 89 NHPIs, 182 Whites, and 13 Others), NHPIs exhibited the highest rates of hypertension (66.3%), diabetes (31.5%), obesity (23.6%), congestive heart failure (13.5%), and coronary artery disease (6.7%). Whites exhibited a higher prevalence of anxiety (18.1%), cardiac arrhythmia (15.4%), and alcohol use (37.4%) compared to Asians and NHPIs. Females had lower mean MMSE scores compared to males (18.3 ± 7.4 vs. 21.0 ± 6.2, respectively), along with higher rates of anxiety (16.3%), hypertension (62.2%), hyperlipidemia (47.4%), and underweight body mass index (10.8%).

Conclusion: NHPI AD patients in Hawaii face a higher prevalence of diabetes and a greater burden of cardiometabolic disorders compared to other racial groups. White AD patients demonstrate higher rates of anxiety, alcohol consumption, and cardiac arrhythmia compared to Asians and NHPIs. Females with AD had worse cognitive function compared to males.

## Introduction

Previous research has identified significant racial disparities in the prevalence of Alzheimer's disease (AD). African Americans and Hispanics have been shown to have higher rates of AD compared to Whites [1]. Additionally, emerging studies suggest that Asians, Native Hawaiians, and Pacific Islanders (NHPI) may also be disproportionately affected by AD [2-4]. However, data on the incidence and prevalence of AD specifically within the NHPI population remains limited, despite this group being rapidly growing and historically underrepresented [5].

Existing literature often either focuses on inpatient data or aggregates NHPI data with that of other minority groups, such as American Indians and Alaska Natives, potentially distorting the findings [6]. Preliminary evidence indicates that NHPIs may experience neurodegenerative disorders, including AD, mild cognitive impairment, and dementia, at younger ages compared to White and Asian populations [3-4].

The reasons behind these racial disparities in AD are complex and multifaceted. Comorbidities such as diabetes, obesity, and cardiovascular disease are known to elevate the risk of developing AD [7-9]. These conditions can lead to structural brain changes through mechanisms like enhanced inflammatory signaling, neuroinflammation, mitochondrial dysfunction, oxidative stress, and insulin resistance, all of which contribute to the pathogenesis of AD, including neuronal loss, synaptic disconnection, tau hyperphosphorylation, and amyloid-beta accumulation [10-13].

In Hawaii, where NHPIs make up approximately 28% of the population, an estimated 11.7% of the overall population has diabetes. Among those with diabetes, 40.7% are aged 65 and older, and 29% belong to the NHPI population [14-15]. Given the established link between AD, diabetes, and other vascular comorbidities, coupled with the high prevalence of these conditions within the NHPI population, it is plausible that NHPI individuals are at an increased risk for AD [4].

In this single-center, retrospective study, we aim to explore the relationship between AD, diabetes, and other comorbidities across common racial groups in Hawaii to better understand the differences in AD presentation among the NHPI population compared to other racial groups. The findings from this study could help guide the development of more effective, targeted interventions for the NHPI population in the prevention and early diagnosis of AD.

## Materials and methods

We conducted a retrospective review of patient records at a single Memory and Alzheimer’s Center in Honolulu, Hawaii, covering the period from June 2018 to June 2024. Data were extracted from patient charts using the 10th revision of the International Classification of Diseases and Related Health Problems [16] clinical modification diagnostic codes G30.1, G30.2, G30.8, and G30.9 for AD (early onset, late onset, unspecified). All patients diagnosed with AD were included in the study but were excluded if their records lacked available Mini-Mental State Examination (MMSE) or Montreal Cognitive Assessment (MoCA) scores [17-18]. The variables of interest included age at AD diagnosis, sex (male/female), insurance type, self-identified ethnicity/race (Asian, White, NHPI, Other), body mass index (BMI) at AD diagnosis, MMSE/MoCA scores, alcohol use, smoking, anxiety, and vascular comorbidities.

Patients self-reported their ethnocultural racial identities. For patients with multiple self-reported identities, categorization was based on their primary self-reported identity. Vascular comorbidities were categorized into the following groups: hypertension, hyperlipidemia, diabetes, coronary artery disease (CAD), congestive heart failure (CHF), cardiac arrhythmia, and stroke or transient ischemic attack (TIA).

To allow for a fair comparison between patients’ cognitive exams, MoCA scores were all converted to MMSE scores using a validated conversion table by Lawton et al. (2016) [19]. Since MMSE/MoCA exams provide quantitative measures of cognition, only scores obtained within one year of diagnosis were included in the study.

Patient demographic variables were summarized using mean and standard deviation for numerical variables and counts and percentages for categorical variables, respectively. Pearson’s chi-squared test and Fisher’s exact test were used to determine if there were any associations between patient race groups and other categorical variables such as demographics and comorbidities. The Kruskal-Wallis rank-sum test was used to assess differences among multiple groups for continuous variables, such as age, MMSE score, and BMI at the time of diagnosis, while the Wilcoxon rank-sum test was applied to compare two independent groups. P-values less than 0.05 were considered statistically significant. Statistical analyses were performed using R software version 4.4.1 (R Foundation for Statistical Computing, Vienna, Austria).

## Results

Data from 540 patients diagnosed with AD who underwent either the MMSE or the MoCA between June 2018 and June 2024 was analyzed. The cohort included 256 Asians, 182 Whites, 89 NHPIs, and 13 patients categorized as Other (including five Blacks, one American Indian, and seven who specified Other).

Patient characteristics and comorbidities

The age at AD diagnosis varied significantly across racial groups (P < 0.001). Asians were diagnosed at a significantly older age (mean age: 78.8 ± 8.0 years) compared to other racial groups, followed by Whites, then NHPIs. Patients in the Other group were diagnosed at the youngest age, with a mean age of 74.0 ± 10.8 years (Table 1). The BMI at the time of AD diagnosis was significantly higher in NHPIs (mean BMI: 27.1 ± 6.1, P < 0.001) compared to other racial groups.

NHPIs had a higher prevalence of hypertension (66.3%, P = 0.003), type I or II diabetes (31.5%, P < 0.001), CAD (13.5%, P = 0.014), and CHF (6.7%, P = 0.007). Whites exhibited the highest rates of alcohol consumption (37.4%, P < 0.001) and cardiac arrhythmia (15.4%, P < 0.001) among the ethnic-racial groups. The Other group had the highest rates of anxiety (23.1%), followed by Whites (18.1%, P = 0.047). No significant differences were observed among the groups regarding sex, insurance, smoking, stroke or TIA, or hyperlipidemia (Tables 1-2).

**Table 1:** Comparison of patient characteristics by ethnic-racial group BMI: body mass index Values are provided as either mean (M) ± standard deviation (SD) or n (%). Percentages are column percentages. *significant at P < 0.05; **significant at P < 0.001; ^§^P-values are based on the Fisher exact test for categorical variables and on the Kruskal-Wallis test for continuous variables.

Characteristics	Total, N = 540	Native Hawaiian and Pacific Islander, N = 89	Asian, N = 256	White, N = 182	Other Races, N = 13	P-value^§^
Age at diagnosis	77.1 (8.6)	75.7 (8.9)	78.8 (8.0)	75.8 (8.8)	74.0 (10.8)	<0.001^**^
Sex						0.15
Male	188 (34.8%)	25 (28.1%)	86 (33.6%)	74 (40.7%)	3 (23.1%)	
Female	352 (65.2%)	64 (71.9%)	170 (66.4%)	108 (59.3%)	10 (76.9%)	
Insurance						0.24
Medicare	505 (93.5%)	86 (96.6%)	242 (94.5%)	164 (90.1%)	13 (100%)	
Military	6 (1.1%)	0 (0%)	1 (0.4%)	5 (2.8%)	0 (0%)	
Private	29 (5.4%)	3 (3.4%)	13 (5.1%)	13 (7.1%)	0 (0%)	
BMI at diagnosis	24.4 (5.0)	27.1 (6.1)	23.4 (4.3)	24.5 (4.7)	23.8 (4.7)	<0.001^**^
Underweight	46 (8.5%)	3 (3.4%)	31 (12.1%)	12 (6.6%)	0 (0%)	
Normal	274 (50.7%)	36 (40.4%)	135 (52.7%)	94 (51.6%)	9 (69.2%)	
Overweight	158 (29.3%)	29 (32.6%)	68 (26.6%)	58 (31.9%)	3 (23.1%)	
Obese	62 (11.5%)	21 (23.6%)	22 (8.6%)	18 (9.9%)	1 (7.7%)	
Alcohol use	131 (24.3%)	14 (15.7%)	46 (18.0%)	68 (37.4%)	3 (23.1%)	<0.001^**^
Smoking	157 (29.1%)	36 (40.5%)	65 (25.4%)	52 (28.6%)	4 (30.8%)	0.053
Anxiety	74 (13.7%)	12 (13.5%)	26 (10.2%)	33 (18.1%)	3 (23.1%)	0.047^*^

**Table 2:** Comparison of vascular comorbidities by racial group TIA: transient ischemic attack Values are provided as n (%). Percentages are column percentages. *significant at P < 0.05; **significant at P < 0.01; ***significant at P < 0.001; ^§^P-values are based on Pearson’s chi-squared test or Fisher exact test for categorical variables (comorbidities)

Vascular comorbidities	Total, N = 540	Native Hawaiian and Pacific Islander, N = 89	Asian, N = 256	White, N = 182	Other races, N = 13	P-value^§^
Hypertension	319 (59.1%)	59 (66.3%)	165 (64.5%)	88 (48.3%)	7 (53.8%)	0.003^**^
Hyperlipidemia	238 (44.1%)	44 (49.4%)	119 (46.5%)	67 (36.8%)	8 (61.5%)	0.067
Diabetes						<0.001^***^
Prediabetes	31 (5.7%)	3 (3.4%)	16 (6.3%)	9 (5.0%)	3 (23.0%)	
Type I or Type II	116 (2.15%)	28 (31.5%)	69 (27.0%)	15 (8.2%)	4 (30.8%)	
Coronary artery disease	31 (5.7%)	12 (13.5%)	9 (3.5%)	10 (5.5%)	0 (0%)	0.014^*^
Congestive heart failure	10 (1.9%)	6 (6.7%)	1 (3.9%)	3 (1.6%)	0 (0%)	0.007^**^
Cardiac arrhythmia	49 (9.1%)	10 (11.2%)	11 (4.3%)	28 (15.4%)	0 (0%)	<0.001^***^
Stroke or TIA	38 (7.0%)	7 (7.9%)	18 (7.0%)	11 (6.0%)	2 (15.4%)	0.52

Cognitive assessments

There were no significant differences in MMSE scores across the ethnic-racial groups (Table 3). However, females had significantly lower mean MMSE scores than males (18.3 ± 7.4 vs. 21.0 ± 6.2, P < 0.001). Further sex-based analysis revealed that females had significantly higher rates of comorbidities such as anxiety (16.3%, P = 0.021), hypertension (62.2%, P = 0.042), hyperlipidemia (47.4%, P = 0.031), and were more than twice as likely to be underweight (10.8%, P < 0.001) compared to males. In contrast, males had higher rates of alcohol use (35.6%, P < 0.001), smoking (37.8%, P = 0.001), obesity (14.4%, P < 0.001), and CAD (9.6%, P = 0.005) compared to females (Table 4).

**Table 3:** Comparison of ethnicity/race and sex by MMSE score MMSE: Mini-Mental State Examination Values are provided as either Mean (M) ± Standard deviation (SD) or n (%). Percentages are column percentages. *significant at P < 0.001; ^§^P-values are based on the Wilcoxon rank sum test and the Kruskal-Wallis test.

Characteristics		Total, N = 540	MMSE score	P-value^§^
Ethnicity/race	Native Hawaiian and Pacific Islander	89 (16.5%)	18.2 (7.4)	0.083
	Asian	256 (47.4%)	19.4 (6.6)	
	White	182 (33.7%)	19.8 (7.6)	
	Other races	13 (2.4%)	18.5 (7.4)	
Sex	Female	352 (65.2%)	18.5 (7.3)	<0.001^*^
	Male	188 (34.8%)	20.8 (6.5)	

**Table 4:** Comparison of patient characteristics and vascular comorbidities by sex BMI: body mass index, TIA: transient ischemic attack Values are provided as either mean (M) ± standard deviation (SD) or n (%). Percentages are column percentages. *significant at P < 0.05; **significant at P < 0.01; ***significant at P < 0.001; ^§^P-values are based on Pearson’s chi-squared test or Fisher exact test for categorical variables (comorbidities) and on the Wilcoxon rank sum test for continuous variables (age and BMI).

Characteristics	Overall, N = 540	Male, N = 188	Female, N = 352	P-value^§^
Age at diagnosis	77.1 (8.6)	76.4 (9.4)	77.5 (8.2)	0.24
BMI at diagnosis	24.4 (5.0)	25.7 (5.2)	23.7 (4.7)	<0.001^***^
Underweight	46 (8.52%)	8 (4.26%)	38 (10.8%)	
Normal	274 (50.7%)	85 (45.2%)	189 (53.7%)	
Overweight	158 (29.3%)	68 (36.2%)	90 (25.6%)	
Obese	62 (11.5%)	27 (14.4%)	35 (9.94%)	
Alcohol use	131 (24.3%)	67 (35.6%)	64 (18.2%)	<0.001^***^
Smoking	157 (29.1%)	71 (37.8%)	86 (24.4%)	0.001^***^
Anxiety	74 (13.8%)	17 (9.09%)	57 (16.3%)	0.021^*^
Hypertension	319 (59.1%)	100 (53.2%)	219 (62.2%)	0.042^*^
Hyperlipidemia	238 (44.1%)	71 (37.8%)	167 (47.4%)	0.031^*^
Diabetes				0.40
Prediabetes	31 (5.74%)	9 (4.79%)	22 (6.25%)	
Type I or Type II	116 (21.5%)	46 (24.5%)	70 (19.9%)	
Coronary artery disease	31 (5.74%)	18 (9.57%)	13 (3.69%)	0.005^**^
Congestive heart failure	10 (1.85%)	3 (1.60%)	7 (1.99%)	>0.99
Cardiac arrhythmias	49 (9.07%)	21 (11.2%)	28 (7.95%)	0.22
Stroke or TIA	38 (7.04%)	18 (9.57%)	20 (5.68%)	0.092

## Discussion

In this study, differences in patient characteristics, vascular comorbidities, and cognitive function, as measured by MMSE and MoCA scores, across various ethnic-racial groups were investigated. Our findings reveal that NHPIs exhibited higher rates of obesity, hypertension, diabetes (type I or II), and cardiovascular diseases compared to other racial groups. These results support previous research indicating that NHPIs have a higher prevalence of cardiometabolic disorders than other racial groups [20]. Socioeconomic factors may partly explain these disparities, as challenges such as limited access to medical care, nutritious food options, and transportation can lead to lifestyle limitations, including physical inactivity, unhealthy dietary habits, and smoking [21]. The lack of patients’ socioeconomic data in our study limits our ability to assess these relationships. Interestingly, we found that NHPIs had the lowest rates of alcohol use among the ethnic-racial groups studied. Although research on substance use in NHPIs is limited, prior studies have produced mixed findings. For example, data from the National Survey on Drug Use and Health suggested that Asians and NHPIs were the least likely to use alcohol and drugs compared to other ethnic-racial groups [22]. Further research is needed to accurately assess the prevalence of alcohol use among NHPIs.

NHPIs and Whites had a similar, younger age at AD onset, while Asians were diagnosed with AD at an older age. Previous studies, however, have shown that NHPIs are more likely to develop earlier onset of AD (EOAD) compared to other racial groups [4,6]. This could be due to the higher prevalence of vascular and metabolic comorbidities in NHPIs, which can accelerate the onset of AD [10,11,13]. Genetic factors, such as the presence of the apolipoprotein E4 (APOE4) allele, are also known to influence AD development. While the apolipoprotein E2 (APOE2) variant is protective against AD, the APOE4 variant increases the risk [23]. However, it is important to note that the APOE4 allele is more commonly associated with late-onset AD rather than EOAD, and a family history of dementia does not appear to have a significant relationship with EOAD [2,6]. A systematic review by Lim et al. (2020) found no association between the APOE4 allele and dementia among the Chamorros, the indigenous population of Guam, suggesting that further research is needed to explore the prevalence and impact of the APOE4 allele in other NHPI populations [2].

Our study also found that Whites had higher rates of anxiety, cardiac arrhythmia, and alcohol consumption compared to Asians and NHPIs. Prior research supports that Whites tend to consume 2-5 more drinks per month than other ethnic-racial groups in the US, with the highest consumption rates found in the Northeast and Western regions [24]. Elevated alcohol consumption may be linked to the higher prevalence of anxiety disorders and cardiac arrhythmias among Whites [25-26]. One study found that Whites are more than three times as likely to be diagnosed with anxiety and more than four times as likely to be diagnosed with post-traumatic stress disorder compared to Asians [27]. We hypothesize that the higher rates of anxiety observed in Whites could also be influenced by differences in cultural values, as Asians are more likely to identify with collectivist values, which can impact self-esteem and stress responses [28]. Additionally, existing literature suggests that while moderate alcohol consumption may offer some protective effects against cardiovascular diseases, chronic heavy drinking can increase the risk of alcoholic cardiomyopathy and cardiac arrhythmia [26].

No significant differences in MMSE/MoCA scores or sex distribution were found among the racial groups. However, females were found to have worse cognitive function than males, potentially due to higher rates of comorbidities such as anxiety, hypertension, and hyperlipidemia. Additionally, females were more likely to be underweight, suggesting the potential role of malnutrition. These findings align with previous research showing that females tend to have longer life expectancies, which increases the likelihood of developing age-related conditions such as cardiometabolic disorders and AD [29]. Furthermore, a randomized clinical trial by Espeland et al. (2015) demonstrated that older females, particularly those undergoing post-menopausal estrogen replacement therapy, face an elevated risk of dementia and cognitive impairment, suggesting that physiological and hormonal differences may contribute to the increased risk and severity of AD in females [30].

Study limitations

This study has several limitations. First, the sample may not accurately represent the broader AD population in Hawaii. Additionally, as a retrospective, single-center study, the findings may not be generalizable to other settings, particularly those with different ethnic-racial compositions or geographies outside the minority-majority state of Hawaii. Furthermore, patients who self-reported multiple ethnicities and/or races were categorized under a single race according to the Hawaii ethnicity coding guide, which could introduce classification bias. The study also did not differentiate between full and part NHPI individuals, making it unclear whether disparities exist between these subgroups. Moreover, NHPIs are distinct groups with different lifestyles and degrees of assimilation to Western culture, which may result in variations in their comorbidities or their AD presentation that were not captured in this study. Finally, the absence of APOE genetic data is another limitation, as it restricts our ability to investigate potential genetic factors that could explain the observed differences between ethnic-racial groups.

## Conclusions

Our study reinforces that NHPIs with AD exhibit higher rates of cardiometabolic disorders - including obesity, hypertension, diabetes (type I or II), CHF, and CAD - compared to other racial groups. Interestingly, NHPIs reported the lowest rates of alcohol consumption. Socioeconomic factors, such as limited access to healthcare, healthy food, and transportation, likely play a role in these disparities by influencing lifestyle choices and health behaviors. These findings emphasize the need for targeted interventions to address the unique health challenges faced by NHPIs.

Additionally, our data show that Whites had higher rates of anxiety, cardiac arrhythmia, and alcohol use compared to Asians and NHPIs. Females with AD demonstrated worse cognitive function than males, along with higher rates of anxiety, hypertension, hyperlipidemia, and lower BMI. These insights enhance our understanding of how AD presents across different ethnic-racial groups and sexes in Hawaii, particularly within NHPI and White populations, and contribute to the limited existing data on the NHPI communities. Our findings underscore the importance of recognizing ethnicity and race-specific differences in AD patients, which can help healthcare providers and public health officials develop targeted prevention strategies and interventions for at-risk populations. Further research is warranted to explore the genetic factors contributing to AD in the NHPI population.

## References

[1] Matthews KA, Xu W, Gaglioti AH, Holt JB, Croft JB, Mack D, McGuire LC (2019). Racial and ethnic estimates of Alzheimer’s disease and related dementias in the United States (2015-2060) in adults aged ≥65 years. Alzheimers Dement.

[2] Lim S, Mohaimin S, Min D (2020). Alzheimer’s disease and its related dementias among Asian Americans, Native Hawaiians, and Pacific Islanders: a scoping review. J Alzheimers Dis.

[3] Sentell TL, Valcour N, Ahn HJ (2015). High rates of Native Hawaiian and older Japanese adults hospitalized with dementia in Hawai'i. J Am Geriatr Soc.

[4] Smith M, Van N, Roberts A (2021). Alzheimer’s disease and its related dementias among Asian Americans, Native Hawaiians, and Pacific Islanders: a scoping review. Cogn Behav Neurol.

[5] (2024). 6 million people in the U.S. identify as Asian, Native Hawaiian or Pacific Islander. https://www.census.gov/library/stories/2022/05/aanhpi-population-diverse-geographically-dispersed.html.

[6] Panegyres PK, Chen HY, Coalition against Major Diseases (CAMD) (2014). Early-onset Alzheimer’s disease: a global cross-sectional analysis. Eur J Neurol.

[7] Baglietto-Vargas D, Shi J, Yaeger DM, Ager R, LaFerla FM (2016). Diabetes and Alzheimer’s disease crosstalk. Neurosci Biobehav Rev.

[8] Dove A, Shang Y, Xu W, Grande G, Laukka EJ, Fratiglioni L, Marseglia A (2021). The impact of diabetes on cognitive impairment and its progression to dementia. Alzheimers Dement.

[9] Qiu C, Kivipelto M, von Strauss E (2009). Epidemiology of Alzheimer's disease: occurrence, determinants, and strategies toward intervention. Dialogues Clin Neurosci.

[10] Al-Kuraishy HM, Jabir MS, Albuhadily AK, Al-Gareeb AI, Rafeeq MF (2023). The link between metabolic syndrome and Alzheimer disease: a mutual relationship and long rigorous investigation. Ageing Res Rev.

[11] Ahmad W, Ijaz B, Shabbiri K, Ahmed F, Rehman S (2017). Oxidative toxicity in diabetes and Alzheimer's disease: mechanisms behind ROS/ RNS generation. J Biomed Sci.

[12] de la Monte SM (2014). Type 3 diabetes is sporadic Alzheimer׳s disease: mini-review. Eur Neuropsychopharmacol.

[13] Pugazhenthi S, Qin L, Reddy PH (2017). Common neurodegenerative pathways in obesity, diabetes, and Alzheimer's disease. Biochim Biophys Acta Mol Basis Dis.

[14] (2024). Adults with diabetes. https://www.hawaiihealthmatters.org/indicators/index/view?indicatorId=81&localeId=14&localeChartIdxs=1%7C2%7C6.

[15] (2024). Hawaii Census Bureau profile. https://data.census.gov/profile/Hawaii?g=040XX00US15.

[16] (2024). International Statistical Classification of Diseases and Related Health Problems 10th revision. https://icd.who.int/browse10/2016/en.

[17] Folstein MF, Folstein SE, McHugh PR (1975). “Mini-mental state”: a practical method for grading the cognitive state of patients for the clinician. J Psychiatr Res.

[18] Nasreddine ZS, Phillips NA, Bédirian V (2005). The Montreal Cognitive Assessment, MoCA: a brief screening tool for mild cognitive impairment. J Am Geriatr Soc.

[19] Lawton M, Kasten M, May MT (2016). Validation of conversion between mini-mental state examination and montreal cognitive assessment. Mov Disord.

[20] Mau MK, Sinclair K, Saito EP, Baumhofer KN, Kaholokula JK (2009). Cardiometabolic health disparities in native Hawaiians and other Pacific Islanders. Epidemiol Rev.

[21] Kawakami KL, Muneoka S, Burrage RL, Tanoue L, Haitsuka K, Braun KL (2022). The lives of Native Hawaiian elders and their experiences with healthcare: a qualitative analysis. Front Public Health.

[22] Wu LT, Swartz MS, Wu Z, Mannelli P, Yang C, Blazer DG (2012). Alcohol and drug use disorders among adults in emergency department settings in the United States. Ann Emerg Med.

[23] Belloy ME, Andrews SJ, Le Guen Y, Cuccaro M, Farrer LA, Napolioni V, Greicius MD (2023). APOE genotype and Alzheimer disease risk across age, sex, and population ancestry. JAMA Neurol.

[24] Bryant AN, Kim G (2019). Regional and racial/ethnic variations in alcohol consumption among older adults. Aging Ment Health.

[25] Morris EP, Stewart SH, Ham LS (2005). The relationship between social anxiety disorder and alcohol use disorders: a critical review. Clin Psychol Rev.

[26] Day E, Rudd JH (2019). Alcohol use disorders and the heart. Addiction.

[27] Asnaani A, Richey JA, Dimaite R, Hinton DE, Hofmann SG (2010). A cross-ethnic comparison of lifetime prevalence rates of anxiety disorders. J Nerv Ment Dis.

[28] Ai AL, Appel HB, Lee J, Fincham F (2022). Family factors related to three major mental health issues among Asian-Americans nationwide. J Behav Health Serv Res.

[29] Beam CR, Kaneshiro C, Jang JY, Reynolds CA, Pedersen NL, Gatz M (2018). Differences between women and men in incidence rates of dementia and Alzheimer’s disease. J Alzheimers Dis.

[30] Espeland MA, Brinton RD, Hugenschmidt C (2015). Impact of type 2 diabetes and postmenopausal hormone therapy on incidence of cognitive impairment in older women. Diabetes Care.

